# On *m*-polar fuzzy graph structures

**DOI:** 10.1186/s40064-016-3066-8

**Published:** 2016-08-30

**Authors:** Muhammad Akram, Rabia Akmal, Noura Alshehri

**Affiliations:** 1 Department of Mathematics, University of the Punjab, New Campus, Lahore, Pakistan; 2 Department of Mathematics, Faculty of Science, Al-Faisaliah Campus, King Abdulaziz University, Jeddah, Saudi Arabia

**Keywords:** *m*-Polar fuzzy graph structure (*m*-PFGSs), Composition, Cartesian product, Strong product, Cross product, Lexicographic product, Join, Union of two *m*-PFGSs, 03E72, 68R10, 68R05

## Abstract

Sometimes information in a network model is based on multi-agent, multi-attribute, multi-object, multi-polar information or uncertainty rather than a single bit. An *m*-polar fuzzy model is useful for such network models which gives more and more precision, flexibility, and comparability to the system as compared to the classical, fuzzy and bipolar fuzzy models. In this research article, we introduce the notion of *m*-polar fuzzy graph structure and present various operations, including Cartesian product, strong product, cross product, lexicographic product, composition, union and join of *m*-polar fuzzy graph structures. We illustrate these operations by several examples. We also investigate some of their related properties.

## Background

Graph theory have applications in many areas of computer science including data mining, image segmentation, clustering, image capturing, networking. A graph structure, introduced by Sampathkumar (2006), is a generalization of undirected graph which is quite useful in studying some structures including graphs, signed graphs, graphs in which every edge is labeled or colored. A graph structure helps to study the various relations and the corresponding edges simultaneously.

A fuzzy set (Zadeh 1965) is an important mathematical structure to represent a collection of objects whose boundary is vague. Fuzzy models are becoming useful because of their aim in reducing the differences between the traditional models used in engineering and science. Nowadays fuzzy sets are playing a substantial role in chemistry, economics, computer science, engineering, medicine and decision making problems. In 1998, Zhang (1998) generalized the idea of a fuzzy set and gave the concept of bipolar fuzzy set on a given set *X* as a map which associates each element of *X* to a real number in the interval $[-1,1].$ In 2014, Chen et al. (2014) introduced the idea of *m*-polar fuzzy sets as an extension of bipolar fuzzy sets and showed that bipolar fuzzy sets and 2-polar fuzzy sets are cryptomorphic mathematical notions and that we can obtain concisely one from the corresponding one in Chen et al. (2014). The idea behind this is that “multipolar information” (not just bipolar information which corresponds to two-valued logic) exists because data for a real world problem are sometimes from *n* agents $(n\ge 2)$. For example, the exact degree of telecommunication safety of mankind is a point in $[0,1]^n (n\approx 7\times 10^9)$ because different person has been monitored different times. There are many examples such as truth degrees of a logic formula which are based on *n* logic implication operators $(n\ge 2)$, similarity degrees of two logic formula which are based on *n* logic implication operators $(n\ge 2)$, ordering results of a magazine, ordering results of a university and inclusion degrees (accuracy measures, rough measures, approximation qualities, fuzziness measures, and decision preformation evaluations) of a rough set.

Kauffman (1973) gave the definition of a fuzzy graph in 1973 on the basis of Zadeh’s fuzzy relations (Zadeh 1971). Rosenfeld (1975) discussed the idea of fuzzy graph in 1975. Further remarks on fuzzy graphs were given by Bhattacharya (1987). Several concepts on fuzzy graphs were introduced by Mordeson and Nair (2001). Akram *et al.* has discussed and introduced bipolar fuzzy graphs, regular bipolar fuzzy graphs, properties of bipolar fuzzy hypergraphs, bipolar fuzzy graph structures and bipolar fuzzy competition graphs in Akram (2011, (2013), Akram and Dudek (2012), Akram et al. (2013), Akram and Akmal (2016) and Al-Shehrie and Akram (2015). In 2015, Akram and Younas studied certain types of irregular *m*-polar fuzzy graphs in Akram and Younas (2016). Akram and Adeel studied m-polar fuzzy line graphs in Akram and Adeel (2016). Akram and Waseem introduced certain metrics in m-polar fuzzy graphs in Akram and Waseem (2016). Dinesh (2014) introduced the notion of a fuzzy graph structure and discussed some related properties. Akram and Akmal (2016) introduced the concept of bipolar fuzzy graph structures. In this research article, we introduce the notion of *m*-polar fuzzy graph structure and present various operations, including Cartesian product, strong product, cross product, lexicographic product, composition, union and join of *m*-polar fuzzy graph structures. We illustrate these operations by several examples. We also investigate some of their related properties. We have used standard definitions and terminologies in this paper. For other notations, terminologies and applications not mentioned in the paper, the readers are referred to Dinesh and Ramakrishnan (2011), Lee (2000) and Zhang (1994).

## Preliminaries

In this section, we review some basic concepts that are necessary for fully benefit of this paper.

In 1965,  Zadeh (1965) introduced the notion of a fuzzy set as follows.

### **Definition 1**

(Zadeh 1965, 1971) A *fuzzy set*$$\mu $$ in a universe *X* is a mapping $$\mu :X\rightarrow [0,1]$$. A *fuzzy relation* on *X* is a fuzzy set $$\nu $$ in $$X \times X$$. Let $$\mu $$ be a fuzzy set in *X* and $$\nu $$ fuzzy relation on *X*. We call $$\nu $$ is a fuzzy relation on $$\mu $$ if $$\nu (x, y) \le $$$$\min \{\mu (x), \mu (y)\}\, \forall x, y \in X$$.

Recently, Akram and Akmal (2016) applied the concept of bipolar fuzzy sets to graph structures.

### **Definition 2**

(Akram and Akmal 2016) $$\check{G_{b}}=(M,N_{1},N_{2},\ldots ,N_{n})$$ is called a *bipolar fuzzy graph structure*(BFGS) of a graph structure (GS) $$G^*=(U,E_{1},E_{2},\ldots ,E_{n})$$ if $$M=(\mu ^{P}_{M},\mu ^{N}_{M})$$ is a *bipolar fuzzy set onU* and for each $$i=1,2,\ldots ,n,$$$$N_{i}=(\mu ^{P}_{N_i},\mu ^{N}_{N_i})$$ is a *bipolar fuzzy set on*$$E_{i}$$ such that

$$\begin{aligned} \mu ^{P}_{N_{i}}(xy)\le \mu ^{P}_{M}(x)\wedge \mu ^{P}_{M}(y),\quad\mu ^{N}_{N_{i}}(xy)\ge \mu ^{N}_{M}(x)\vee \mu ^{N}_{M}(y) \quad\forall \,xy\in E_{i}\subset U\times U. \end{aligned}$$

Note that $$\mu ^{P}_{N_{i}}(xy)=0=\mu ^{N}_{N_{i}}(xy)$$ for all $$xy \in U \times U -E_i$$ and $$0 < \mu ^{P}_{N_{i}}(xy) \le 1$$, $$-1 \le \mu ^{N}_{N_{i}}(xy) < 0$$$$\forall \,xy\in E_{i},$$ where *U* and $$E_i\,(i=1,2,\ldots ,n)$$ are called *underlying vertex set* and *underlying i-edge sets* of $$\check{G_b}$$, respectively.

### **Definition 3**

(Akram and Akmal 2016) Let $\check{G_{b}}=(M,N_{1},N_{2},\ldots ,N_{n})$ be a BFGS of a *GS*$G^*=(U,E_{1},E_{2},\ldots ,E_{n}).$ Let $\phi$ be any permutation on the set $\{E_{1},E_{2},\ldots ,E_{n}\}$ and the corresponding permutation on $\{N_{1},N_{2},\ldots ,N_{n}\},$ i.e., $\phi (N_{i})=N_{j}$ if and only if $\phi (E_{i})=E_{j}\,\forall i.$

If $xy\in N_{r}$ for some *r* and

$$\begin{aligned}&\mu ^P_{N^\phi _{i}}(xy)=\mu ^P_M(x)\wedge \mu ^P_M(y)-\bigvee \limits _{j\ne i}\mu ^P_{\phi N_{j}}(xy),\\&\mu ^{N}_{N^\phi _{i}}(xy)=\mu ^N_M(x)\vee \mu ^N_M(y)-\bigwedge \limits _{j\ne i}\mu ^N_{\phi N_{j}}(xy),\quad i=1,2,\ldots ,n, \end{aligned}$$

then $xy\in B^\phi _{m},$ while *m* is chosen such that $\mu ^P_{N^\phi _{m}}(xy)\ge \mu ^P_{N^\phi _{i}} (xy)\,and\,\mu ^{N}_{N^\phi _{m}}(xy)\le \mu ^{N}_{N^\phi _{i}}(xy)\,\forall i.$

And BFGS $(M,{N^\phi _{1}},{N^\phi _{2}},\ldots ,{N^\phi _{n}})$ denoted by $\check{G}^{\phi c}_{b},$ is called the $\phi$-*complement of BFGS*$\check{G_{b}}.$

Chen et al. (2014) introduced the notion of *m*-polar fuzzy set as a generalization of a bipolar fuzzy set.

### **Definition 4**

(Chen et al. 2014) An *m-polar fuzzy set* (or a $[0,1]^m$-set) on *X* is exactly a mapping $A:X\rightarrow [0,1]^m.$

Note that $[0, 1]^m$ (*m*th-power of [0, 1]) is considered as a poset with the point-wise order $\le$, where *m* is an arbitrary ordinal number (we make an appointment that $m= \{n | n < m \}$ when $m>0$), $\le$ is defined by $x \le y\Leftrightarrow p_i(x) \le p_i(y)$ for each $i \in m$ ( $x, y \in [0, 1]^m)$, and $p_i : [0, 1]^m \rightarrow [0, 1]$ is the *i*th projection mapping $(i \in m)$. ${\mathbf 0}=(0,0,\ldots , 0)$ is the smallest element in $[0,1]^m$ and ${\mathbf 1}=(1,1,\ldots ,1)$ is the largest element in $[0,1]^m$. Akram and Waseem (2016) defined *m*-polar fuzzy relation as follows.

### **Definition 5**

(Akram and Waseem 2016) Let *C* be an *m*-polar fuzzy subset of a non-empty set *V*. An *m-polar fuzzy relation* on *C* is an *m*-polar fuzzy subset *D* of $V\times V$ defined by the mapping $D:V\times V\rightarrow [0,1]^m$ such that for all $x,\,y\in V,p_i\circ D(xy)\le \inf \{p_i\circ C(x),p_i\circ C(y)\},\,i=1,2,\ldots ,m,$ where $p_i\circ C(x)$ denotes the *i*th degree of membership of the vertex *x* and $p_i\circ D(xy)$ denotes the *i*th degree of membership of the edge *xy*.

An *m*-polar fuzzy graph was introduced by Chen et al. (2014) and modified by Akram and Waseem (2016).

### **Definition 6**

(Akram and Waseem 2016), Chen et al. (2014) An *m-polar fuzzy graph* is a pair $G=(C,D)$, where $C:V\rightarrow [0,1]^m$ is an *m*-polar fuzzy set in *V* and $D: V \times V\rightarrow [0,1]^m$ is an *m*-polar fuzzy relation on *V* such that

$$\begin{aligned} p_i\circ D(xy)\le \inf \{p_i\circ C(x),p_i\circ C(y)\} \end{aligned}$$

for all $$x,y\in V.$$

We note that $p_i\circ D(xy)=0$ for all $xy\in V\times V-E$ for all $i=1,2,3,\ldots ,m.$*C* is called the *m-polar fuzzy vertex set* of *G* and *D* is called the *m-polar fuzzy edge set* of *G*,  respectively. An *m*-polar fuzzy relation *D* on *V* is called symmetric if $p_i\circ D(xy)=p_i\circ D(yx)$ for all $x,y\in V.$

## *m*-Polar fuzzy graph structures

We first define the concept of an *m*-polar fuzzy graph structure.

### **Definition 7**

Let $G^*=(U,E_1,E_2,\ldots ,E_n)$ be a graph structure (GS). Let *C* be an *m*-polar fuzzy set on *U* and $D_i$ an *m*-polar fuzzy set on $E_i$ such that

$$\begin{aligned}p_j\circ D_i(xy)\le \inf \{p_j\circ C(x),p_j\circ C(y)\}\end{aligned}$$

for all $$x,y\in U$$, $$i\in n,$$$$j\in m$$ and $$p_j\circ D_i(xy)=0$$ for $$xy\in U\times U{\setminus } E_i,$$$$\forall j$$. Then $$G_{(m)}=(C,D_1,D_2,\ldots ,D_n)$$ is called an *m*-*polar fuzzy graph structure* (*m*-PFGS) on $$G^*$$ where *C* is the *m*-*polar fuzzy vertex set* of $$G_{(m)}$$ and $$D_i$$ is the *m*-*polar fuzzyi-edge set* of $$G_{(m)}$$.

We illustrate the concept of an *m*-polar fuzzy graph structure with an example.

### *Example 8*

Consider a graph structure $G^*=(U,E_{1},E_{2})$ such that $U=\{a_1,a_2,a_3,a_4\}$, $E_1=\{a_1a_2\}$ and $E_2=\{a_3a_2,a_2a_4\}.$ Let *C*, $D_1$ and $D_2$ be 4-polar fuzzy sets on $U,\,E_1$ and $E_2$, respectively, defined by the following tables:

**Table:** 

*C*	$a_1$	$a_2$	$a_3$	$a_4$
$p_1\circ C$	0.1	0.3	0.4	0.2
$p_2\circ C$	0.0	0.6	0.0	0.0
$p_3\circ C$	0.0	0.2	0.4	0.3
$p_4\circ C$	0.1	0.0	0.4	0.4

**Table:** 

$D_i$	$(a_1a_2)_1$	$(a_3a_2)_2$	$(a_2a_4)_2$	
$p_1\circ D_i$	0.1	0.2	0.2	
$p_2\circ D_i$	0.0	0.0	0.0	
$p_3\circ D_i$	0.0	0.2	0.2	
$p_4\circ D_i$	0.0	0.0	0.0	

**Fig. 1:**
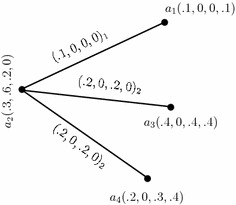
4-Polar fuzzy graph structure

By simple calculations, it is easy to check that $G_{(m)}=(C,D_{1},D_{2})$ is a 4-polar fuzzy graph structure of $G^*$ as shown in Fig. 1. Note that we represent $xy\in D_i$ as $(xy)_i=(p_1\circ D_i(xy),\ldots ,p_m\circ D_i(xy))_i$ in all tables and the figures.

Note that operations on *m*-polar fuzzy sets are generalization of operations on bipolar fuzzy sets. We apply the concept of *m*-polar fuzzy sets on some operations of graph structures.

### **Definition 9**

Let $G^1_{(m)}=(C_1,D_{11},D_{12},\ldots ,D_{1n})$ and $G^2_{(m)}=(C_2,D_{21},D_{22},\ldots ,D_{2n})$ be two *m-PFGSs*. Then the *Cartesian product* of $G^1_{(m)}$ and $G^2_{(m)}$ is given by

$$\begin{aligned} G^1_{(m)}\times G^2_{(m)}=(C_1\times C_2,D_{11}\times D_{21},D_{12}\times D_{22},\ldots ,D_{1n}\times D_{2n}) \end{aligned}$$

where the mappings $C_1\times C_2:U_1\times U_2\rightarrow [0,1]^m$ and $D_{1i}\times D_{2i}:E_{1i}\times E_{2i}\rightarrow [0,1]^m$ (for $i\in n$) are respectively defined by

$$\begin{aligned} p_j\circ (C_1\times C_2)(x_1x_2)=p_j\circ C_1(x_1)\wedge p_j\circ C_2(x_2),\quad \forall \,x_1x_2\in U_1\times U_2 \end{aligned}$$

and

$$\begin{aligned} p_j\circ (D_{1i}\times &  D_{2i})((xx_2)(xy_2))=p_j\circ C_{1}(x)\wedge p_j\circ D_{2i}(x_2y_2),\quad \forall x\in U_1,\,x_2y_2\in E_{2i},\\ p_j\circ (D_{1i}\times &  D_{2i})((x_1y)(y_1y))=p_j\circ C_{2}(y)\wedge p_j\circ D_{1i}(x_1y_1),\quad \forall y\in U_2,\,x_1y_1\in E_{1i}, \end{aligned}$$

where *j* varies from 1 to *m*.

We illustrate Cartesian product of $G^1_{(m)}$ and $G^2_{(m)}$ with an example.

### *Example 10*

**Fig. 2:**
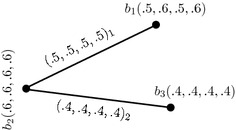
4-Polar fuzzy graph structure

Let $$G^1_{(m)}=(C',D'_{1},D'_{2})$$ be a 4-PFGS of graph structure $$G^*_1=(U',E'_{1},E'_{2})$$ where $$U'=\{b_1,b_2,b_3\},\,E'_{1}=\{b_1b_2\}$$ and $$E'_{2}=\{b_2b_3\}.$$$$G^1_{(m)}$$ is drawn and shown in the Fig. 2.

**Fig. 3:**
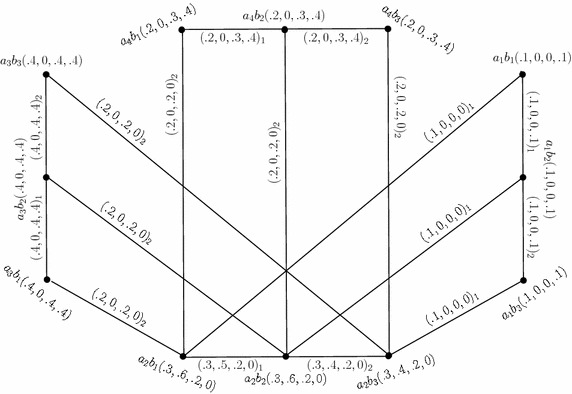
Cartesian product of two 4-PFGSs

The Cartesian product of $G_{(m)}$ (Fig. 1) and $G^1_{(m)},$ given by $G_{(m)}\times G^1_{(m)}=(C\times C',D_{1}\times D'_{1},D_{2}\times D'_{2}),$ is as shown in Fig. 3. In the figure, a $D_{i}\times D'_{i}$-edge can be identified by the subscript “*i*” with the corresponding degrees of memberships of edge.

We now formulate Cartesian product of $G^1_{(m)}$ and $G^2_{(m)}$ as a proposition.

### **Proposition 11**

*Cartesian product of twom-polar fuzzy graph structures is anm-polar fuzzy graph structure.*

### *Proof*

Let GS $G^*=(U_1\times U_2,E_{11}\times E_{21},E_{12}\times E_{22},\ldots ,E_{1n}\times E_{2n})$ be the Cartesian product of GSs $G^*_1=(U_1,E_{11},E_{12},\ldots ,E_{1n})$ and $G^*_2=(U_2,E_{21},E_{22},\ldots ,E_{2n}).$ Let $G^1_{(m)}=(C_1,D_{11},D_{12},\ldots ,D_{1n})$ and $G^2_{(m)}=(C_2,D_{21},D_{22},\ldots ,D_{2n})$ be respective *m*-PFGSs of $G^*_1$ and $G^*_2.$ Then $(C_1\times C_2,D_{11}\times D_{21},D_{12}\times D_{22},\ldots ,D_{1n}\times D_{2n})$ is an *m*-PFGS of $G^*.$By the Definition 9 of Cartesian product, $C_1\times C_2$ is an *m*-polar fuzzy set of $U_1\times U_2$ and $D_{1i}\times D_{2i}$ is an *m*-polar fuzzy set of $E_{1i}\times E_{2i}$ for all *i*. So the remaining task is to prove that $D_{1i}\times D_{2i}$ is an *m*-polar fuzzy relation on $C_1\times C_2$ for all *i*. For this, some cases are discussed, as follows:

**Case 1**. When $x\in U_1$ and $x_2y_2\in E_{2i}$

$$\begin{aligned}&p_j\circ (D_{1i}\times D_{2i})((xx_2)(x y_2))\\&= p_j\circ C_{1}(x)\wedge p_j\circ D_{2i}(x_2y_2) \\&\le p_j\circ C_{1}(x)\wedge [\inf \{p_j\circ C_2(x_2),\,p_j\circ C_2(y_2)\}] \\&= \inf \{p_j\circ C_{1}(x)\wedge p_j\circ C_2(x_2),\,p_j\circ C_{1}(x)\wedge p_j\circ C_2(y_2)\} \\&= \inf \{p_j\circ (C_1\times C_2)(xx_2),\,p_j\circ (C_1\times C_2)(x y_2)\}, \quad \forall j\in m. \end{aligned}$$

**Case 2**. When $$y\in U_2,\,x_1y_1\in E_{1i}$$

$$\begin{aligned}&p_j\circ (D_{1i}\times D_{2i})((x_1y)(y_1y))\\&= p_j\circ C_{2}(y)\wedge p_j\circ D_{1i}(x_1y_1) \\&\le p_j\circ C_{2}(y)\wedge [\inf \{p_j\circ C_1(x_1),\,p_j\circ C_1(y_1)\}] \\&= \inf \{p_j\circ C_{2}(y)\wedge p_j\circ C_1(x_1),\,p_j\circ C_{2}(y)\wedge p_j\circ C_1(y_1)\} \\&= \inf \{p_j\circ C_1(x_1)\wedge p_j\circ C_{2}(y),\,p_j\circ C_1(y_1)\wedge p_j\circ C_{2}(y)\} \\&= \inf \{p_j\circ (C_1\times C_2)(x_1y),\,p_j\circ (C_1\times C_2)(y_1y)\}, \quad \forall j\in m. \end{aligned}$$

Both cases hold for every $i\in n.$ This completes the proof.□

We define cross product of $G^1_{(m)}$ and $G^2_{(m)}$ by an example.

### **Definition 12**

Let $G^1_{(m)}=(C_1,D_{11},D_{12},\ldots ,D_{1n})$ and $G^2_{(m)}=(C_2,D_{21},D_{22},\ldots ,D_{2n})$ be two *m-PFGSs*. Then the *cross product* of $G^1_{(m)}$ and $G^2_{(m)}$ is given by

$$\begin{aligned} G^1_{(m)}* G^2_{(m)}=(C_1* C_2,D_{11}* D_{21},D_{12}* D_{22},\ldots ,D_{1n}* D_{2n}) \end{aligned}$$

where the mappings $C_1*C_2:U_1* U_2\rightarrow [0,1]^m$ and $D_{1i}* D_{2i}:E_{1i}* E_{2i}\rightarrow [0,1]^m$ (for $i\in n$) are respectively defined by

$$\begin{aligned} p_j\circ (C_1* C_2)(x_1x_2)=p_j\circ C_1(x_1)\wedge p_j\circ C_2(x_2),\quad \forall \,x_1x_2\in U_1* U_2=U_1\times U_2 \end{aligned}$$

and

$$\begin{aligned} p_j\circ (D_{1i}* D_{2i})((x_1x_2)(y_1y_2))=p_j\circ D_{1i}(x_1y_1)\wedge p_j\circ D_{2i}(x_2y_2),\quad \forall x_1y_1\in E_{1i},\,x_2y_2\in E_{2i}, \end{aligned}$$

where *j* varies from 1 to *m*.

We explain the concept of cross product of two *m*-polar fuzzy graph structures with an example.

### *Example 13*

**Fig. 4:**
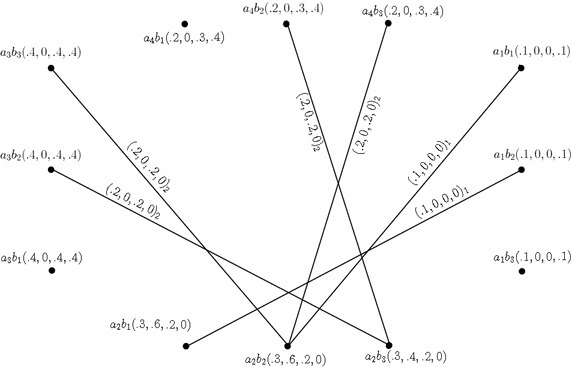
Cross product of two 4-PFGSs

Consider the 4-PFGSs $G_{(m)}$ and $G^1_{(m)}$ shown in the Figs. 1 and 2, respectively. The cross product of $G_{(m)}$ and $G^1_{(m)},$ given by $G_{(m)}* G^1_{(m)}=(C*C',D_{1}* D'_{1},D_{2}*D'_{2}),$ is as shown in Fig. 4. In the figure, a $D_{i}*D'_{i}$-edge can be identified by the subscript “*i*” with the corresponding degrees of memberships of edge.

We formulate cross product of two *m*-polar fuzzy graph structures as a proposition.

### **Proposition 14**

*Cross product of two m-polar fuzzy graph structures is anm-polar fuzzy graph structure.*

### *Proof*

Let GS $G^*=(U_1* U_2,E_{11}* E_{21},E_{12}* E_{22},\ldots ,E_{1n}* E_{2n})$ be the cross product of GSs $G^*_1=(U_1,E_{11},E_{12},\ldots ,E_{1n})$ and $G^*_2=(U_2,E_{21},E_{22},\ldots ,E_{2n}).$ If $G^1_{(m)}=(C_1,D_{11},D_{12},\ldots ,D_{1n})$ and $G^2_{(m)}=(C_2,D_{21},D_{22},\ldots ,D_{2n})$ are respective *m*-PFGSs of $G^*_1$ and $G^*_2$ then $(C_1* C_2,D_{11}* D_{21},D_{12}* D_{22},\ldots ,D_{1n}*D_{2n})$ is an *m*-PFGS of $G^*.$ By the Definition 12 of cross product, $C_1* C_2$ and $D_{1i}* D_{2i}$ are *m*-polar fuzzy sets of $U_1* U_2$ and $E_{1i}* E_{2i}$, respectively, for all *i*. So remaining task is to prove that $D_{1i}* D_{2i}$ is an *m*-polar fuzzy relation on $C_1* C_2$ for all *i*. For this, proceed as follows:

If $x_1y_1\in E_{1i}$ and $x_2y_2\in E_{2i}$, then

$$\begin{aligned}&p_j\circ (D_{1i}* D_{2i})((x_1x_2)(y_1y_2))\\&= p_j\circ D_{1i}(x_1y_1)\wedge p_j\circ D_{2i}(x_2y_2) \\&\le [\inf \{p_j\circ C_1(x_1),\,p_j\circ C_1(y_1)\}]\wedge [\inf \{p_j\circ C_2(x_2),\,p_j\circ C_2(y_2)\}] \\&= \inf \{p_j\circ C_{1}(x_1)\wedge p_j\circ C_2(x_2),\,p_j\circ C_{1}(y_1)\wedge p_j\circ C_2(y_2)\} \\&= \inf \{p_j\circ (C_1* C_2)(x_1x_2),\,p_j\circ (C_1* C_2)(y_1y_2)\}, \quad \forall j\in m. \end{aligned}$$

This holds for every $i\in n.$ Hence $D_{1i}* D_{2i}$ is an *m*-polar fuzzy relation on $C_1* C_2$, for all *i*, which completes the proof. $\square$

We now define lexicographic product of *m*-polar fuzzy graph structures.

### **Definition 15**

Let $G^1_{(m)}=(C_1,D_{11},D_{12},\ldots ,D_{1n})$ and $G^2_{(m)}=(C_2,D_{21},D_{22},\ldots ,D_{2n})$ be two *m-PFGSs*. Then the *lexicographic product* of $G^1_{(m)}$ and $G^2_{(m)}$ is given by

$$\begin{aligned} G^1_{(m)}\bullet G^2_{(m)}=(C_1\bullet C_2,D_{11}\bullet D_{21},D_{12}\bullet D_{22},\ldots ,D_{1n}\bullet D_{2n}) \end{aligned}$$

where the mappings $C_1\bullet C_2:U_1\bullet U_2\rightarrow [0,1]^m$ and $D_{1i}\bullet D_{2i}:E_{1i}\bullet E_{2i}\rightarrow [0,1]^m$ (for $i\in n$) are respectively defined by

$$\begin{aligned} p_j\circ (C_1\bullet C_2)(x_1x_2)=p_j\circ C_1(x_1) \wedge p_j\circ C_2(x_2),\quad \forall \,x_1x_2\in U_1\bullet U_2=U_1\times U_2 \end{aligned}$$

and

$$\begin{aligned}&p_j\circ (D_{1i}\bullet D_{2i})((xx_2)(xy_2))=p_j\circ C_{1}(x)\wedge p_j\circ D_{2i}(x_2y_2),\, \forall x\in U_1,\,x_2y_2\in E_{2i},\\&\quad p_j\circ (D_{1i}\bullet D_{2i})((x_1x_2)(y_1y_2))=p_j\circ D_{1i}(x_1y_1)\wedge p_j\circ D_{2i}(x_2y_2),\quad \forall x_1y_1\in E_{1i},\,x_2y_2\in E_{2i}, \end{aligned}$$

where *j* varies from 1 to *m*.

We explain the concept of lexicographic product of *m*-polar fuzzy graph structures by the following example.

### *Example 16*

**Fig. 5:**
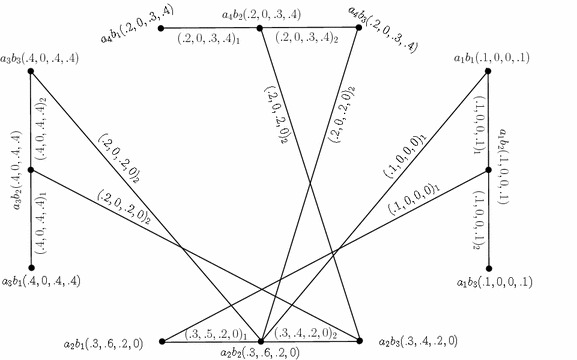
Lexicographic product of two 4-PFGSs

Consider the 4-PFGSs $G_{(m)}$ and $G^1_{(m)}$ shown in the Figs. 1 and 2, respectively. The lexicographic product of $G_{(m)}$ and $G^1_{(m)},$ given by $G_{(m)}\bullet G^1_{(m)}=(C\bullet C',D_{1}\bullet D'_{1},D_{2}\bullet D'_{2}),$ is as shown in Fig. 5. In the figure, a $D_{i}\bullet D'_{i}$-edge can be identified by the subscript “*i*” with the corresponding degrees of memberships of edge.

We formulate Lexicographic product of two *m*-polar fuzzy graph structures as a proposition.

### **Proposition 17**

*Lexicographic product of two m-polar fuzzy graph structures is anm-polar fuzzy graph structure.*

### *Proof*

Let GS $G^*=(U_1\bullet U_2,E_{11}\bullet E_{21},E_{12}\bullet E_{22},\ldots ,E_{1n}\bullet E_{2n})$ be the lexicographic product of GSs $G^*_1=(U_1,E_{11},E_{12},\ldots ,E_{1n})$ and $G^*_2=(U_2,E_{21},E_{22},\ldots ,E_{2n}).$ If $G^1_{(m)}=(C_1,D_{11},D_{12},\ldots ,D_{1n})$ and $G^2_{(m)}=(C_2,D_{21},D_{22},\ldots ,D_{2n})$ are respective *m*-PFGSs of $G^*_1$ and $G^*_2$ then $(C_1\bullet C_2,D_{11}\bullet D_{21},D_{12}\bullet D_{22},\ldots ,D_{1n}\bullet D_{2n})$ is an *m*-PFGS of $G^*.$ By the Definition 15 of lexicographic product, $C_1\bullet C_2$ and $D_{1i}\bullet D_{2i}$ are *m*-polar fuzzy sets of $U_1\bullet U_2$ and $E_{1i}\bullet E_{2i}$, respectively, for all *i*. Now, remaining task is to prove that $D_{1i}\bullet D_{2i}$ is an *m*-polar fuzzy relation on $C_1\bullet C_2$ for all *i*. For this, we discuss two cases as follows:

**Case 1**. When $x\in U_1$ and $x_2y_2\in E_{2i}$

$$\begin{aligned}&p_j\circ (D_{1i}\bullet D_{2i})((xx_2)(x y_2))\\&= p_j\circ C_{1}(x)\wedge p_j\circ D_{2i}(x_2y_2) \\&\le p_j\circ C_{1}(x)\wedge [\inf \{p_j\circ C_2(x_2),\,p_j\circ C_2(y_2)\}] \\&= \inf \{p_j\circ C_{1}(x)\wedge p_j\circ C_2(x_2),\,p_j\circ C_{1}(x)\wedge p_j\circ C_2(y_2)\} \\&= \inf \{p_j\circ (C_1\bullet C_2)(xx_2),\,p_j\circ (C_1\bullet C_2)(x y_2)\}, \quad \forall j\in m. \end{aligned}$$

**Case 2**. When $x_1y_1\in E_{1i}$ and $x_2y_2\in E_{2i}$,

$$\begin{aligned}&p_j\circ (D_{1i}\bullet D_{2i})((x_1x_2)(y_1y_2))\\&= p_j\circ D_{1i}(x_1y_1)\wedge p_j\circ D_{2i}(x_2y_2) \\&\le [\inf \{p_j\circ C_1(x_1),\,p_j\circ C_1(y_1)\}]\wedge [\inf \{p_j\circ C_2(x_2),\,p_j\circ C_2(y_2)\}] \\&= \inf \{p_j\circ C_{1}(x_1)\wedge p_j\circ C_2(x_2),\,p_j\circ C_{1}(y_1)\wedge p_j\circ C_2(y_2)\} \\&= \inf \{p_j\circ (C_1\bullet C_2)(x_1x_2),\,p_j\circ (C_1\bullet C_2)(y_1y_2)\},\quad \forall j\in m. \end{aligned}$$

This holds for every $i\in n.$ Hence $D_{1i}\bullet D_{2i}$ is an *m*-polar fuzzy relation on $C_1\bullet C_2$, for all *i*, which completes the proof. $\square$

We now give definition of strong product of *m*-polar fuzzy graph structures.

### **Definition 18**

Let $G^1_{(m)}=(C_1,D_{11},D_{12},\ldots ,D_{1n})$ and $G^2_{(m)}=(C_2,D_{21},D_{22},\ldots ,D_{2n})$ be two *m-PFGSs*. Then the *strong product* of $G^1_{(m)}$ and $G^2_{(m)}$ is given by

$$\begin{aligned} G^1_{(m)}\boxtimes G^2_{(m)}=(C_1\boxtimes C_2,D_{11}\boxtimes D_{21},D_{12}\boxtimes D_{22},\ldots ,D_{1n}\boxtimes D_{2n})\end{aligned}$$

where the mappings $C_1\boxtimes C_2:U_1\boxtimes U_2\rightarrow [0,1]^m$ and $D_{1i}\boxtimes D_{2i}:E_{1i}\boxtimes E_{2i}\rightarrow [0,1]^m$ (for $i\in n$) are respectively defined by

$$\begin{aligned} p_j\circ (C_1\boxtimes C_2)(x_1x_2)=p_j\circ C_1(x_1)\wedge p_j\circ C_2(x_2),\quad \forall \,x_1x_2\in U_1\boxtimes U_2=U_1\times U_2 \end{aligned}$$

and

$$\begin{aligned}&p_j\circ (D_{1i}\boxtimes D_{2i})((xx_2)(xy_2))=p_j\circ C_{1}(x)\wedge p_j\circ D_{2i}(x_2y_2),\quad \forall x\in U_1,\,x_2y_2\in E_{2i},\\& p_j\circ (D_{1i}\boxtimes D_{2i})((x_1y)(y_1y))=p_j\circ C_{2}(y)\wedge p_j\circ D_{1i}(x_1y_1),\quad \forall y\in U_2,\,x_1y_1\in E_{1i},\\& p_j\circ (D_{1i}\boxtimes D_{2i})((x_1x_2)(y_1y_2))=p_j\circ D_{1i}(x_1y_1)\wedge p_j\circ D_{2i}(x_2y_2),\quad \forall x_1y_1\in E_{1i},\,x_2y_2\in E_{2i}, \end{aligned}$$

where *j* varies from 1 to *m*.

We illustrate the idea of strong product of *m*-polar fuzzy graph structures by the following example.

### *Example 19*

**Fig. 6:**
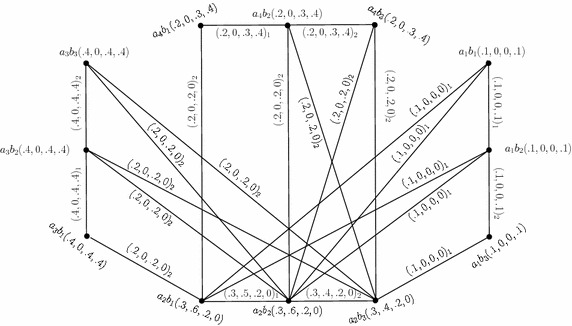
Strong product of two 4-PFGSs

Consider the 4-PFGSs $G_{(m)}$ and $G^1_{(m)}$ shown in the Figs. 1 and 2, respectively. The strong product of $G_{(m)}$ and $G^1_{(m)},$ given by $G_{(m)}\boxtimes G^1_{(m)}=(C\boxtimes C',D_{1}\boxtimes D'_{1},D_{2}\boxtimes D'_{2}),$ is as shown in Fig. 6. In the figure, a $D_{i}\boxtimes D'_{i}$-edge can be identified by the subscript “*i*” with the corresponding degrees of memberships of edge.

We formulate strong product of $G^1_{(m)}$ and $G^2_{(m)}$ as a proposition.

### **Proposition 20**

*Strong product of two m-polar fuzzy graph structures is anm-polar fuzzy graph structure.*

### *Proof*

Let GS $G^*=(U_1\boxtimes U_2,E_{11}\boxtimes E_{21},E_{12}\boxtimes E_{22},\ldots ,E_{1n}\boxtimes E_{2n})$ be the strong product of GSs $G^*_1=(U_1,E_{11},E_{12},\ldots ,E_{1n})$ and $G^*_2=(U_2,E_{21},E_{22},\ldots ,E_{2n}).$ Let $G^1_{(m)}=(C_1,D_{11},D_{12},\ldots ,D_{1n})$ and $G^2_{(m)}=(C_2,D_{21},D_{22},\ldots ,D_{2n})$ be respective *m*-PFGSs of $G^*_1$ and $G^*_2.$ Then $(C_1\boxtimes C_2,D_{11}\boxtimes D_{21},D_{12}\boxtimes D_{22},\ldots ,D_{1n}\boxtimes D_{2n})$ is an *m*-PFGS of $G^*.$ By Definition 18 of strong product, $C_1\boxtimes C_2$ is an *m*-polar fuzzy set of $U_1\boxtimes U_2$ and $D_{1i}\boxtimes D_{2i}$ is an *m*-polar fuzzy set of $E_{1i}\boxtimes E_{2i}$ for all *i*. So the remaining task is to prove that $D_{1i}\boxtimes D_{2i}$ is an *m*-polar fuzzy relation on $C_1\boxtimes C_2$ for all *i*. For this, some cases are discussed, as follows:

**Case 1**. When $x\in U_1$ and $x_2y_2\in E_{2i}$

$$\begin{aligned}&p_j\circ (D_{1i}\boxtimes D_{2i})((xx_2)(x y_2))\\&\quad = p_j\circ C_{1}(x)\wedge p_j\circ D_{2i}(x_2y_2) \\&\quad \le p_j\circ C_{1}(x)\wedge [\inf \{p_j\circ C_2(x_2),\,p_j\circ C_2(y_2)\}] \\&\quad = \inf \{p_j\circ C_{1}(x)\wedge p_j\circ C_2(x_2),\,p_j\circ C_{1}(x)\wedge p_j\circ C_2(y_2)\} \\&\quad = \inf \{p_j\circ (C_1\boxtimes C_2)(xx_2),\,p_j\circ (C_1\boxtimes C_2)(x y_2)\},\quad \forall j\in m. \end{aligned}$$

**Case 2**. When $$y\in U_2,\,x_1y_1\in E_{1i}$$

$$\begin{aligned}&p_j\circ (D_{1i}\boxtimes D_{2i})((x_1y)(y_1y))\\&= p_j\circ C_{2}(y)\wedge p_j\circ D_{1i}(x_1y_1) \\& \le p_j\circ C_{2}(y)\wedge [\inf \{p_j\circ C_1(x_1),\,p_j\circ C_1(y_1)\}] \\& = \inf \{p_j\circ C_{2}(y)\wedge p_j\circ C_1(x_1),\,p_j\circ C_{2}(y)\wedge p_j\circ C_1(y_1)\} \\& = \inf \{p_j\circ C_1(x_1)\wedge p_j\circ C_{2}(y),\,p_j\circ C_1(y_1)\wedge p_j\circ C_{2}(y)\} \\& = \inf \{p_j\circ (C_1\boxtimes C_2)(x_1y),\,p_j\circ (C_1\boxtimes C_2)(y_1y)\},\quad \forall j\in m. \end{aligned}$$

**Case 3**. When $x_1y_1\in E_{1i}$ and $x_2y_2\in E_{2i}$,

$$\begin{aligned}&p_j\circ (D_{1i}\boxtimes D_{2i})((x_1x_2)(y_1y_2))\\&\quad = p_j\circ D_{1i}(x_1y_1)\wedge p_j\circ D_{2i}(x_2y_2) \\&\quad \le [\inf \{p_j\circ C_1(x_1),\,p_j\circ C_1(y_1)\}]\wedge [\inf \{p_j\circ C_2(x_2),\,p_j\circ C_2(y_2)\}] \\&\quad = \inf \{p_j\circ C_{1}(x_1)\wedge p_j\circ C_2(x_2),\,p_j\circ C_{1}(y_1)\wedge p_j\circ C_2(y_2)\} \\&\quad = \inf \{p_j\circ (C_1\boxtimes C_2)(x_1x_2),\,p_j\circ (C_1\boxtimes C_2)(y_1y_2)\},\quad \forall j\in m. \end{aligned}$$

All three cases hold for every $i\in n.$ This completes the proof. $\square$

We define the notion of composition of two *m*-polar fuzzy graph structures.

### **Definition 21**

Let $G^1_{(m)}=(C_1,D_{11},D_{12},\ldots ,D_{1n})$ and $G^2_{(m)}=(C_2,D_{21},D_{22},\ldots ,D_{2n})$ be two *m-PFGSs*. Then *composition* of $G^1_{(m)}$ and $G^2_{(m)}$ is given by

$$\begin{aligned} G^1_{(m)}\circ G^2_{(m)}=(C_1\circ C_2,D_{11}\circ D_{21},D_{12}\circ D_{22},\ldots ,D_{1n}\circ D_{2n}) \end{aligned}$$

where the mappings $C_1\circ C_2:U_1\circ U_2\rightarrow [0,1]^m$ and $D_{1i}\circ D_{2i}:E_{1i}\circ E_{2i}\rightarrow [0,1]^m$ (for $i\in n$) are respectively defined by

$$\begin{aligned} p_j\circ (C_1\circ C_2)(x_1x_2)=p_j\circ C_1(x_1) \wedge p_j\circ C_2(x_2),\quad \forall \,x_1x_2\in U_1\circ U_2=U_1\times U_2 \end{aligned}$$

and

$$\begin{aligned}&p_j\circ (D_{1i}\circ D_{2i})((xx_2)(xy_2))=p_j\circ C_{1}(x)\wedge p_j\circ D_{2i}(x_2y_2),\quad \forall x\in U_1,\,x_2y_2\in E_{2i},\\&\quad p_j\circ (D_{1i}\circ D_{2i})((x_1y)(y_1y))=p_j\circ C_{2}(y)\wedge p_j\circ D_{1i}(x_1y_1),\quad \forall y\in U_2,\,x_1y_1\in E_{1i},\\&\quad p_j\circ (D_{1i}\circ D_{2i})((x_1x_2)(y_1y_2))  =p_j\circ D_{1i}(x_1y_1)\wedge p_j\circ C_{2}(x_2)\wedge p_j\circ C_{2}(y_2),\quad \forall x_1y_1\in E_{1i},x_2,y_2\in U_{2},\,{\text{such that}} \,x_2\ne y_2, \end{aligned}$$

where *j* varies from 1 to *m*.

We discuss the notion of composition of two *m*-polar fuzzy graph structures by the following example.

### *Example 22*

**Fig. 7:**
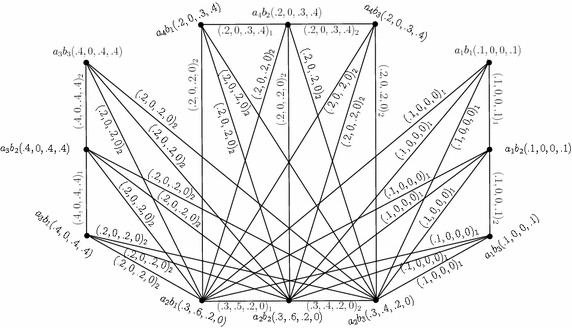
Composition of two 4-PFGSs

Consider the 4-PFGSs $G_{(m)}$ and $G^1_{(m)}$ shown in the Fig. 1 and The composition of $G_{(m)}$ and $G^1_{(m)},$ given by $G_{(m)}\circ G^1_{(m)}=(C\circ C',D_{1}\circ D'_{1},D_{2}\circ D'_{2}),$ is as shown in Fig. 7. In the figure, a $D_{i}\circ D'_{i}$-edge can be identified by the subscript “*i*” with the corresponding degrees of memberships of edge.

We present composition of two *m*-polar fuzzy graph structures as a propostion.

### **Proposition 23**

*Composition of two m-polar fuzzy graph structures is anm-polar fuzzy graph structure.*

### *Proof*

Let GS $G^*=(U_1\circ U_2,E_{11}\circ E_{21},E_{12}\circ E_{22},\ldots ,E_{1n}\circ E_{2n})$ be the composition of GSs $G^*_1=(U_1,E_{11},E_{12},\ldots ,E_{1n})$ and $G^*_2=(U_2,E_{21},E_{22},\ldots ,E_{2n}).$ Let $G^1_{(m)}=(C_1,D_{11},D_{12},\ldots ,D_{1n})$ and $G^2_{(m)}=(C_2,D_{21},D_{22},\ldots ,D_{2n})$ be respective *m*-PFGSs of $G^*_1$ and $G^*_2.$ Then $(C_1\circ C_2,D_{11}\circ D_{21},D_{12}\circ D_{22},\ldots ,D_{1n}\circ D_{2n})$ is an *m*-PFGS of $G^*.$ By Definition 21 of composition, $C_1\circ C_2$ is an *m*-polar fuzzy set of $U_1\circ U_2$ and $D_{1i}\circ D_{2i}$ is an *m*-polar fuzzy set of $E_{1i}\circ E_{2i}$ for all *i*. Therefore the remaining task is to show that $D_{1i}\circ D_{2i}$ is an *m*-polar fuzzy relation on $C_1\circ C_2$ for all *i*. For this, consider the following cases:

**Case 1**. When $x\in U_1$ and $x_2y_2\in E_{2i}$

$$\begin{aligned}&p_j\circ (D_{1i}\circ D_{2i})((xx_2)(x y_2))\\&\quad = p_j\circ C_{1}(x)\wedge p_j\circ D_{2i}(x_2y_2) \\&\quad \le p_j\circ C_{1}(x)\wedge [\inf \{p_j\circ C_2(x_2),\,p_j\circ C_2(y_2)\}] \\&\quad = \inf \{p_j\circ C_{1}(x)\wedge p_j\circ C_2(x_2),\,p_j\circ C_{1}(x)\wedge p_j\circ C_2(y_2)\} \\&\quad = \inf \{p_j\circ (C_1\circ C_2)(xx_2),\,p_j\circ (C_1\circ C_2)(x y_2)\},\quad \forall j\in m. \end{aligned}$$

**Case 2**. When $$y\in U_2,\,x_1y_1\in E_{1i}$$

$$\begin{aligned}&p_j\circ (D_{1i}\circ D_{2i})((x_1y)(y_1y))\\&\quad = p_j\circ C_{2}(y)\wedge p_j\circ D_{1i}(x_1y_1) \\&\quad \le p_j\circ C_{2}(y)\wedge [\inf \{p_j\circ C_1(x_1),\,p_j\circ C_1(y_1)\}] \\&\quad = \inf \{p_j\circ C_{2}(y)\wedge p_j\circ C_1(x_1),\,p_j\circ C_{2}(y)\wedge p_j\circ C_1(y_1)\} \\&\quad = \inf \{p_j\circ C_1(x_1)\wedge p_j\circ C_{2}(y),\,p_j\circ C_1(y_1)\wedge p_j\circ C_{2}(y)\} \\&\quad = \inf \{p_j\circ (C_1\circ C_2)(x_1y),\,p_j\circ (C_1\circ C_2)(y_1y)\},\quad \forall j\in m. \end{aligned}$$

**Case 3**. When $x_1y_1\in E_{1i}$ and $x_2,y_2\in U_{2}$, such that $x_2\ne y_2$,

$$\begin{aligned}&p_j\circ (D_{1i}\circ D_{2i})((x_1x_2)(y_1y_2))\\&\quad = p_j\circ D_{1i}(x_1y_1)\wedge p_j\circ C_{2}(x_2)\wedge p_j\circ C_{2}(y_2) \\&\quad \le [\inf \{p_j\circ C_1(x_1),\,p_j\circ C_1(y_1)\}]\wedge p_j\circ C_{2}(x_2)\wedge p_j\circ C_{2}(y_2) \\&\quad = \inf \{[p_j\circ C_{1}(x_1)\wedge p_j\circ C_2(x_2)\wedge p_j\circ C_{2}(y_2)],\,[p_j\circ C_{1}(y_1)\wedge p_j\circ C_2(x_2)\wedge p_j\circ C_2(y_2)]\} \\&\quad \le \inf \{[p_j\circ C_{1}(x_1)\wedge p_j\circ C_2(x_2)],\,[p_j\circ C_{1}(y_1)\wedge p_j\circ C_2(y_2)]\} \\&\quad = \inf \{p_j\circ (C_1\circ C_2)(x_1x_2),\,p_j\circ (C_1\circ C_2)(y_1y_2)\},\quad \forall j\in m. \end{aligned}$$

All three cases hold for every $i\in n.$ This completes the proof.□

We now introduce the concept of union of two *m*-polar fuzzy graph structures.

### **Definition 24**

Let $G^1_{(m)}=(C_1,D_{11},D_{12},\ldots ,D_{1n})$ and $G^2_{(m)}=(C_2,D_{21},D_{22},\ldots ,D_{2n})$ be two *m-PFGSs*. Then *union* of $G^1_{(m)}$ and $G^2_{(m)}$ is given by

$$\begin{aligned} G^1_{(m)}\cup G^2_{(m)}=(C_1\cup C_2,D_{11}\cup D_{21},D_{12}\cup D_{22},\ldots ,D_{1n}\cup D_{2n}) \end{aligned}$$

where the mappings $C_1\cup C_2:U_1\cup U_2\rightarrow [0,1]^m$ and $D_{1i}\cup D_{2i}:E_{1i}\cup E_{2i}\rightarrow [0,1]^m$ (for $i\in n$) are respectively defined by

$$\begin{aligned} p_j\circ (C_1\cup C_2)(x)=\left\{ \,\begin{array}{l} p_j\circ C_1(x),\quad \forall x\in U_1{\setminus } U_2\\ p_j\circ C_2(x),\quad \forall x\in U_2{\setminus } U_1\\ p_j\circ C_1(x)\vee p_j\circ C_2(x),\quad \forall x\in U_1\cap U_2 \end{array}\right. \end{aligned}$$

and

$$\begin{aligned} p_j\circ (D_{1i}\cup D_{2i})(x_1x_2)=\left\{ \begin{array}{l} p_j\circ D_{1i}(x_1x_2),\quad \forall x_1x_2\in E_{1i}{\setminus } E_{2i} \\ p_j\circ D_{2i}(x_1x_2),\quad \forall x_1x_2\in E_{2i}{\setminus } E_{1i} \\ p_j\circ D_{1i}(x_1x_2)\vee p_j\circ D_{2i}(x_1x_2),\quad \forall x_1x_2\in E_{1i}\cap E_{2i} \end{array}\right. \end{aligned}$$

where *j* varies from 1 to *m*.

We describe the concept of union of two *m*-polar fuzzy graph structures with an example.

### *Example 25*

**Fig. 8:**
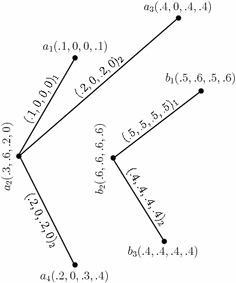
Union of two *m*-PFGSs

Consider the 4-PFGSs $G_{(m)}$ and $G^1_{(m)}$ shown in the Figs. 1 and 2, respectively. The union of $G_{(m)}$ and $G^1_{(m)},$ given by $G_{(m)}\cup G^1_{(m)}=(C\cup C',D_{1}\cup D'_{1},D_{2}\cup D'_{2}),$ is as shown in Fig. 8. In the figure, a $D_{i}\cup D'_{i}$-edge can be identified by the subscript “*i*” with the corresponding degrees of memberships of edge.

### **Proposition 26**

*Union of two m-polar fuzzy graph structures is anm-polar fuzzy graph structure.*

### *Proof*

Let GS $G^*=(U_1\cup U_2,E_{11}\cup E_{21},E_{12}\cup E_{22},\ldots ,E_{1n}\cup E_{2n})$ be the union of GSs $G^*_1=(U_1,E_{11},E_{12},\ldots ,E_{1n})$ and $G^*_2=(U_2,E_{21},E_{22},\ldots ,E_{2n}).$ Let $G^1_{(m)}=(C_1,D_{11},D_{12},\ldots ,D_{1n})$ and $G^2_{(m)}=(C_2,D_{21},D_{22},\ldots ,D_{2n})$ be respective *m*-PFGSs of $G^*_1$ and $G^*_2.$ Then $(C_1\cup C_2,D_{11}\cup D_{21},D_{12}\cup D_{22},\ldots ,D_{1n}\cup D_{2n})$ is an *m*-PFGS of $G^*.$ From the Definition 24 of union, $C_1\cup C_2$ is an *m*-polar fuzzy set of $U_1\cup U_2$ and $D_{1i}\cup D_{2i}$ is an *m*-polar fuzzy set of $E_{1i}\cup E_{2i}$ for all *i*. So the remaining task is to show that $D_{1i}\cup D_{2i}$ is an *m*-polar fuzzy relation on $C_1\cup C_2$ for all *i*. For this, consider following cases:

**Case 1**. When $x_1x_2\in E_{1i}{\setminus } E_{2i}$, then there are three possibilities (i) $x_1,x_2\in U_1$ (ii) $x_1\in U_1,x_2\in U_1\cap U_2$ (ii) $x_2\in U_1,x_1\in U_1\cap U_2$. So for all $j\in m$

$$\begin{aligned}&p_j\circ (D_{1i}\cup D_{2i})(x_1x_2)\\&= p_j\circ D_{1i}(x_1x_2) \\& \le \inf \{p_j\circ C_1(x_1),\,p_j\circ C_1(x_2)\}\\& = \inf \{p_j\circ (C_1\cup C_2)(x_1),p_j\circ (C_1\cup C_2)(x_2)\},\quad{\mathrm{if}}\,x_1,x_2\in U_1.\\& \le \inf [p_j\circ C_1(x_1),\max \{p_j\circ C_1(x_2),p_j\circ C_2(x_2)\}]\\& = \inf \{p_j\circ (C_1\cup C_2)(x_1),p_j\circ (C_1\cup C_2)(x_2)\},\quad{\mathrm{if}}\,x_1\in U_1,x_2\in U_1\cap U_2.\\& \le \inf [\max \{p_j\circ C_1(x_1),p_j\circ C_2(x_1)\},p_j\circ C_1(x_2)]\\&= \inf \{p_j\circ (C_1\cup C_2)(x_1),p_j\circ (C_1\cup C_2)(x_2)\},\quad{\mathrm{if}}\,x_2\in U_1,x_1\in U_1\cap U_2. \end{aligned}$$

**Case 2**. When $x_1x_2\in E_{2i}{\setminus } E_{1i}$, then there are three possibilities (i) $x_1,x_2\in U_2$ (ii) $x_1\in U_2,x_2\in U_1\cap U_2$ (ii) $x_2\in U_2,x_1\in U_1\cap U_2$. So for all $j\in m$

$$\begin{aligned}&p_j\circ (D_{1i}\cup D_{2i})(x_1x_2)\\& = p_j\circ D_{2i}(x_1x_2)\\& \le \inf \{p_j\circ C_2(x_1),\,p_j\circ C_2(x_2)\}\\& = \inf \{p_j\circ (C_1\cup C_2)(x_1),p_j\circ (C_1\cup C_2)(x_2)\},\quad{\mathrm{if}}\,x_1,x_2\in U_2.\\& \le \inf [p_j\circ C_2(x_1),\max \{p_j\circ C_1(x_2),p_j\circ C_2(x_2)\}]\\& = \inf \{p_j\circ (C_1\cup C_2)(x_1),p_j\circ (C_1\cup C_2)(x_2)\},\quad{\mathrm{if}}\,x_1\in U_2,x_2\in U_1\cap U_2.\\& \le \inf [\max \{p_j\circ C_1(x_1),p_j\circ C_2(x_1)\},p_j\circ C_2(x_2)]\\& = \inf \{p_j\circ (C_1\cup C_2)(x_1),p_j\circ (C_1\cup C_2)(x_2)\},\quad{\mathrm{if}}\,x_2\in U_2,x_1\in U_1\cap U_2. \end{aligned}$$

**Case 3**. When $x_1x_2\in E_{2i}\cap E_{1i}$, then $x_1,x_2\in U_1\cap U_2$. So

$$\begin{aligned}&p_j\circ (D_{1i}\cup D_{2i})(x_1x_2)\\&\quad = [p_j\circ D_{1i}(x_1x_2)]\vee [p_j\circ D_{2i}(x_1x_2)] \\&\quad \le [\inf \{p_j\circ C_1(x_1),\,p_j\circ C_1(x_2)\}]\vee [\inf \{p_j\circ C_2(x_1),\,p_j\circ C_2(x_2)\}] \\&\quad =\inf [\inf \{p_j\circ C_1(x_1),\,p_j\circ C_1(x_2)\}\vee \{p_j\circ C_2(x_1)\},\inf \{p_j\circ C_1(x_1),\,p_j\circ C_1(x_2)\}\vee \{p_j\circ C_2(x_2)\}] \\&\quad \le \inf [\{p_j\circ C_1(x_1)\}\vee \{p_j\circ C_2(x_1)\},\{p_j\circ C_1(x_2)\}\vee \{p_j\circ C_2(x_2)\}] \\&\quad=\inf [p_j\circ (C_1\cup C_2)(x_1),\,p_j\circ (C_1\cup C_2)(x_2)],\quad \forall j\in m. \end{aligned}$$

All three cases hold for every $i\in n.$ Hence $D_{1i}\cup D_{2i}$ is an *m*-polar fuzzy relation on $C_1\cup C_2$ for all *i*. This completes the proof. $\square$

### **Theorem 27**

*If GS*$G^*=(U_1\cup U_2,E_{11}\cup E_{21},E_{12}\cup E_{22},\ldots ,E_{1n}\cup E_{2n})$* is the union of GSs*$G^*_1=(U_1,E_{11},E_{12},\ldots ,E_{1n})$* and*$G^*_2=(U_2,E_{21},E_{22},\ldots ,E_{2n}).$* Then everym*-PFGS $(C,D_1,D_2,\ldots ,D_n)$* of*$G^*$*is the union of an m-PFGS*$G^1_{(m)}$* of*$G^*_1$*and an m-PFGS*$G^2_{(m)}$* of*$G^*_2.$

### *Proof*

Observe that $$C=C_1\cup C_2$$, $$D_i=D_{1i}\cup D_{2i}$$ and $$C_1,$$$$C_2,$$$$D_{1i}$$ and $$D_{2i}$$ are *m*-polar fuzzy sets on $$U_1,$$$$U_2,$$$$E_{1i}$$ and $$E_{2i}$$, respectively, for $$i\in n$$ if for every *j*, we define $$C_1,$$$$C_2,$$$$D_{1i}$$ and $$D_{2i}$$ as:

$$\begin{aligned} p_j\circ C_1(x)& =  p_j\circ C(x),\quad {\mathrm{if}}\,u\in U_1{\setminus } U_2.\\ p_j\circ C_2(x)& =  p_j\circ C(x),\quad {\mathrm{if}}\,u\in U_2{\setminus } U_1.\\ p_j\circ C_1(x)& =  p_j\circ C_2(x)=p_j\circ C(x),\quad {\mathrm{if}}\,u\in U_2\cap U_1.\\ p_j\circ D_{1i}(x_1x_2)& =  p_j\circ D_{i}(x_1x_2),\quad {\mathrm{if}}\,(x_1x_2)\in E_{1i}{\setminus } E_{2i}.\\ p_j\circ D_{2i}(x_1x_2)& =  p_j\circ D_{i}(x_1x_2),\quad {\mathrm{if}}\,(x_1x_2)\in E_{2i}{\setminus } E_{1i}.\\ p_j\circ D_{1i}(x_1x_2)& =  p_j\circ D_{2i}(x_1x_2)=p_j\circ D_{i}(x_1x_2),\quad {\mathrm{if}}\,(x_1x_2)\in E_{1i}\cap E_{2i}. \end{aligned}$$

For $k=1,2$, $D_{ki}$ is an *m*-polar fuzzy relation on $C_k$, since

$$\begin{aligned} p_j\circ D_{ki}(x_1x_2)=p_j\circ D_{i}(x_1x_2)\le \inf \{p_j\circ C(x_1),\,p_j\circ C(x_2)\}=\inf \{p_j\circ C_k(x_1),\,p_j\circ C_k(x_2)\}. \end{aligned}$$

Therefore, $G^k_{(m)}=(C_k,D_{k1},\ldots ,D_{kn})$ is a *m*-PFGS of $G^*_k$ for $k=1,2$ and *m*-PFGS $(C,D_1,\ldots ,D_n)$ is union of *m*-PFGS $G^1_{(m)}=(C_1,D_{11},D_{12},\ldots ,D_{1n})$ and *m*-PFGS $G^2_{(m)}=(C_2,D_{21},D_{22},\ldots ,D_{2n})$. Hence every *m*-PFGS of $G^*=\bigcup \nolimits _k G^*_{k},$ is the union of some *m*-PFGSs of $G^*_k$ for $k=1,2.$□

Finally, we study the concept of join of two *m*-polar fuzzy graph structures.

### **Definition 28**

Let $G^1_{(m)}=(C_1,D_{11},D_{12},\ldots ,D_{1n})$ and $G^2_{(m)}=(C_2,D_{21},D_{22},\ldots ,D_{2n})$ be two *m-PFGSs* such that $U_1\cap U_2=\emptyset$. Let $U_{1i}=\{x\in U_1:All\,the\,edges\,incident\,with\,x\,are\,E_{1i}-edges\}$ and $U_{2i}=\{x\in U_2:All\,the\,edges\,incident\,with\,x\,are\,E_{2i}-edges\}$. Then *join* of $G^1_{(m)}$ and $G^2_{(m)}$ is given by

$$\begin{aligned} G^1_{(m)}+ G^2_{(m)}=(C_1+ C_2,D_{11}+ D_{21},D_{12}+ D_{22},\ldots ,D_{1n}+ D_{2n}) \end{aligned}$$

where the mappings $C_1+ C_2:U_1+ U_2\rightarrow [0,1]^m$ and $D_{1i}+ D_{2i}:E_{1i}+ E_{2i}\rightarrow [0,1]^m$ (for $i\in n$) are respectively defined by

$$\begin{aligned} p_j\circ (C_1+ C_2)(x)=\left\{ \begin{array}{l} p_j\circ C_1(x),\quad \forall x\in U_1 \\ p_j\circ C_2(x),\quad \forall x\in U_2 \end{array}\right. \end{aligned}$$

and

$$\begin{aligned} \,p_j\circ (D_{1i}+ D_{2i})(x_1x_2)=\left\{ \begin{array}{l} p_j\circ D_{1i}(x_1x_2),\quad \forall x_1x_2\in E_{1i} \\ p_j\circ D_{2i}(x_1x_2),\quad \forall x_1x_2\in E_{2i} \\ \inf \{p_j\circ C_1(x_1),p_j\circ C_2(x_2)\},\quad \forall x_1\in U_{1i},\,x_2\in U_{2i} \,\end{array}\right. \end{aligned}$$

where *j* varies from 1 to *m*.

### *Example 29*

**Fig. 9:**
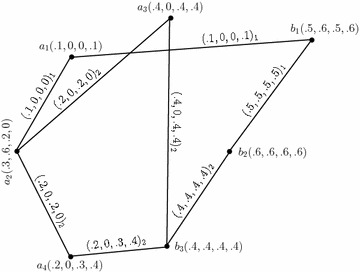
Join of two *m*-PFGSs

Consider the 4-PFGSs $G_{(m)}$ and $G^1_{(m)}$ shown in the Figs. 1 and 2, respectively. The join of $G_{(m)}$ and $G^1_{(m)},$ given by $G_{(m)}+ G^1_{(m)}=(C+ C',D_{1}+ D'_{1},D_{2}+ D'_{2}),$ is as shown in Fig. 9. In the figure, a $D_{i}+ D'_{i}$-edge can be identified by the subscript “*i*” with the corresponding degrees of memberships of edge.

### **Proposition 30**

*Let GS*$G^*=(U_1+ U_2,E_{11}+ E_{21},E_{12}+ E_{22},\ldots ,E_{1n}+ E_{2n})$* be the join of GSs*$G^*_1=(U_1,E_{11},E_{12},\ldots ,E_{1n})$* and*$G^*_2=(U_2,E_{21},E_{22},\ldots ,E_{2n}).$* Let*$G^1_{(m)}=(C_1,D_{11},D_{12},\ldots ,D_{1n})$* and*$G^2_{(m)}=(C_2,D_{21},D_{22},\ldots ,D_{2n})$*be respective m*-PFGSs of $G^*_1$* and*$G^*_2.$* Then*$(C_1+ C_2,D_{11}+ D_{21},D_{12}+ D_{22},\ldots ,D_{1n}+ D_{2n})$*is an m*-PFGS of $G^*.$

### *Proof*

From the Definition 28 of Join, $C_1+ C_2$ is an *m*-polar fuzzy set of $U_1+ U_2$ and $D_{1i}+ D_{2i}$ is an *m*-polar fuzzy set of $E_{1i}+ E_{2i}$ for all *i*. So the remaining task is to show that $D_{1i}+ D_{2i}$ is an *m*-polar fuzzy relation on $C_1+ C_2$ for all *i*. For this, consider following cases:

**Case 1**. When $x_1x_2\in E_{1i}$, then $x_1,x_2\in U_1$. So

$$\begin{aligned}&p_j\circ (D_{1i}+ D_{2i})(x_1x_2)\\&\quad = p_j\circ D_{1i}(x_1x_2) \\&\quad \le \inf \{p_j\circ C_1(x_1),\,p_j\circ C_1(x_2)\} \\&\quad = \inf \{p_j\circ (C_1+ C_2)(x_1),\,p_j\circ (C_1+ C_2)(x_2)\},\quad \forall j\in m. \end{aligned}$$

**Case 2**. When $x_1x_2\in E_{2i}$, then $x_1,x_2\in U_2$. So

$$\begin{aligned}&p_j\circ (D_{1i}+ D_{2i})(x_1x_2)\\&\quad = p_j\circ D_{2i}(x_1x_2) \\&\quad \le \inf \{p_j\circ C_2(x_1),\,p_j\circ C_2(x_2)\} \\&\quad = \inf \{p_j\circ (C_1+ C_2)(x_1),\,p_j\circ (C_1+ C_2)(x_2)\},\quad \forall j\in m. \end{aligned}$$

**Case 3**. When $x_1\in U_{1i},\,x_2\in U_{2i}$, then $x_1\in U_1,\,x_2\in U_2$. So

$$\begin{aligned}&p_j\circ (D_{1i}+ D_{2i})(x_1x_2)\\&\quad= [p_j\circ C_1(x_1)]\wedge [p_j\circ C_2(x_2)] \\&\quad = [p_j\circ (C_1+ C_2)(x_1)]\wedge [p_j\circ (C_1+ C_2)(x_2)] \\&\quad =\inf [p_j\circ (C_1+ C_2)(x_1),\,p_j\circ (C_1+ C_2)(x_2)],\quad \forall j\in m. \end{aligned}$$

Hence $D_{1i}+ D_{2i}$ is an *m*-polar fuzzy relation on $C_1+ C_2$ in all three cases. All cases hold for every $i\in n.$ This completes the proof. $\square$

### **Theorem 31**

*If GS*$G^*=(U_1+ U_2,E_{11}+ E_{21},E_{12}+ E_{22},\ldots ,E_{1n}+ E_{2n})$* is the join of GSs*$G^*_1=(U_1,E_{11},E_{12},\ldots ,E_{1n})$* and*$G^*_2=(U_2,E_{21},E_{22},\ldots ,E_{2n}).$* Then every strongm*-PFGS $(C,D_1,D_2,\ldots ,D_n)$* of*$G^*$*is the join of a strong m-PFGS of*$G^*_1$* and a strongm*-PFGS of $G^*_2.$□

### *Proof*

Let $$(C,D_1,D_2,\ldots ,D_n)$$ be a strong *m*-PFGS of $$G^*$$. Define $$C_1,$$$$C_2,$$$$D_{1i}$$ and $$D_{2i}$$ for every *j*,  as follows:

$$\begin{aligned} p_j\circ C_1(x)& =  p_j\circ C(x),\quad {\mathrm{if}}\,u\in U_1,\\ p_j\circ C_2(x)& =  p_j\circ C(x),\quad {\mathrm{if}}\,u\in U_2,\\ p_j\circ D_{1i}(x_1x_2)& =  p_j\circ D_{i}(x_1x_2),\quad {\mathrm{if}}\,(x_1x_2)\in E_{1i},\\ p_j\circ D_{2i}(x_1x_2)& =  p_j\circ D_{i}(x_1x_2),\quad {\mathrm{if}}\,(x_1x_2)\in E_{2i}. \end{aligned}$$

Observe that $$C_1,$$$$C_2,$$$$D_{1i}$$ and $$D_{2i}$$ are *m*-polar fuzzy sets on $$U_1,$$$$U_2,$$$$E_{1i}$$ and $$E_{2i}$$, respectively, for $$i\in n$$. For $$k=1,2$$, $$D_{ki}$$ is an *m*-polar fuzzy relation on $$C_k$$, so $$G^k_{(m)}=(C_k,D_{k1},\ldots ,D_{kn})$$ is a strong *m*-PFGS of $$G^*_k,$$ since

$$\begin{aligned} p_j\circ D_{ki}(x_1x_2)=p_j\circ D_{i}(x_1x_2)= \inf \{p_j\circ C(x_1),\,p_j\circ C(x_2)\}=\inf \{p_j\circ C_k(x_1),\,p_j\circ C_k(x_2)\} \end{aligned}$$

for all $x_1x_2\in E_{ki}$. Moreover, $C=C_1+C_2$ and $D_i=D_{1i}+D_{2i}$, since $p_j\circ D_i(x_1x_2)=p_j\circ (D_{1i}+D_{2i})(x_1x_2)$ for all $x_1x_2\in E_{1i}\cup E_{2i}$ and $p_j\circ D_i(x_1x_2)=\inf \{p_j\circ C(x_1),\,p_j\circ C(x_2)\}=\inf \{p_j\circ C_{1}(x_1),\,p_j\circ C_{2})(x_2)\}=p_j\circ (D_{1i}+D_{2i})(x_1x_2)$ for all $x_1\in U_{1i},x_2\in U_{2i}.$ Therefore *m*-PFGS $(C,D_1,\ldots ,D_n)$ is join of *m*-PFGS $G^1_{(m)}=(C_1,D_{11},D_{12},\ldots ,D_{1n})$ and *m*-PFGS $G^2_{(m)}=(C_2,D_{21},D_{22},\ldots ,D_{2n})$. Hence a strong *m*-PFGS of $G^*=G^*_{1}+G^*_{2}$ is the join of a strong *m*-PFGSs of $G^*_1$ and a strong *m*-PFGSs of $G^*_2$. Which completes the proof. $\square$

## Conclusions

A graph structure is a useful tool in solving the combinatorial problems in different areas of computer science and computational intelligence systems. It helps to study various relations and the corresponding edges simultaneously. We have introduced the notion of *m*-polar fuzzy graph structure, and presented various methods of their construction. We are extending our work to (1) domination in bipolar fuzzy graph structure, (2) bipolar fuzzy soft graph structures, (3) roughness in graph structures, (4) intuitionistic fuzzy soft graph structures, and (5) multiple-attribute decision making methods based on *m*-polar fuzzy graph structures.
